# Lasers in Live Cell Microscopy

**DOI:** 10.3390/ijms23095015

**Published:** 2022-04-30

**Authors:** Herbert Schneckenburger

**Affiliations:** Institute of Applied Research, Aalen University, 73430 Aalen, Germany; herbert.schneckenburger@hs-aalen.de

**Keywords:** confocal microscopy, light sheet microscopy, TIRFM, super-resolution, spectral imaging, FLIM, FRET, optical tweezers, laser micromanipulation, photodynamic therapy

## Abstract

Due to their unique properties—coherent radiation, diffraction limited focusing, low spectral bandwidth and in many cases short light pulses—lasers play an increasing role in live cell microscopy. Lasers are indispensable tools in 3D microscopy, e.g., confocal, light sheet or total internal reflection microscopy, as well as in super-resolution microscopy using wide-field or confocal methods. Further techniques, e.g., spectral imaging or fluorescence lifetime imaging (FLIM) often depend on the well-defined spectral or temporal properties of lasers. Furthermore, laser microbeams are used increasingly for optical tweezers or micromanipulation of cells. Three exemplary laser applications in live cell biology are outlined. They include fluorescence diagnosis, in particular in combination with Förster Resonance Energy Transfer (FRET), photodynamic therapy as well as laser-assisted optoporation, and demonstrate the potential of lasers in cell biology and—more generally—in biomedicine.

## 1. Introduction

Since their development by Theodore Maiman in 1960, lasers represent a class of light sources in the visible, near-ultraviolet or near/middle infrared spectral range, which are based on the stimulated emission of radiation. Due to this principle, lasers possess specific and unique properties regarding coherence, tunability, focusing and creation of short light pulses, which often makes them indispensable tools in biomedical optics. Laser materials include gases, liquids (dyes) and various kinds of solids, e.g., solid matrices doped with rare earth materials, semiconductors or optical fibers. A summary of the most convenient continuous wave (cw) and pulsed lasers are given in Table 1. Most of these lasers are operated at one or several discrete wavelengths either in their basic mode or at multiple frequencies (e.g., frequency doubling or tripling), resulting in shorter wavelengths. Tunable lasers within a broader spectral range include dye lasers, titanium: sapphire lasers or super-continuum fiber lasers.

In comparison with conventional light sources, lasers offer numerous advantages in live cell research, e.g., light microscopy. Laser light is coherent in general, i.e., the light wave has a well-defined phase, which can be used for quantitative phase imaging [1] and interference studies. Optical coherence tomography (OCT) is a well-known method for tissue diagnostics, which is based on the interference of two laser beams within well-defined layers, which can be shifted in a three-dimensional specimen for tomographic imaging. Meanwhile, this method has been extended from living tissue to single cells [2,3,4]. Further methods of phase imaging including holography [5,6,7] and ptychography [8,9] have been applied in the label-free microscopy of living cells, and phase contrast, as well as interference contrast microscopy, are used to make the phase visible in transmission microscopy. In the two latter cases, however, low coherence in the micrometer range, as provided by conventional light sources is usually sufficient, and lasers are not necessary. Light interference is also used in Structured Illumination Microscopy (SIM) [10,11], a method of super-resolution microscopy permitting a two-fold better resolution in comparison with the so-called Abbe criterion. A main advantage of SIM compared to other super-resolution methods is that light exposure is rather low, and only very little phototoxic damage to living specimens is expected to occur.

A further advantage of lasers is their low spectral bandwidth. This does not only permit excitation of relevant fluorophores in the maximum of their absorption bands but also measurement of inelastic light scattering, e.g., Raman scattering [12,13,14], at wavelengths that are rather close to the excitation wavelength. Monochromatic laser light can also be modulated electro- or acoustically in order to generate ultra-short pulses, which are used for fluorescence lifetime imaging microscopy or measurement of Förster Resonance Energy Transfer (FRET) [15,16,17] between adjacent molecules.

In contrast to conventional light sources, lasers can be focused to a diffraction limited spot whose diameter is often in the sub-micrometer range or, if a cylindrical instead of a spherical lens is used, to a light sheet, as frequently applied in Light Sheet Fluorescence Microscopy (LSFM) [18,19] to selectively illuminate single planes of a 3D specimen. LSFM is an alternative method to Confocal Laser Scanning Microscopy (CLSM) with low light exposure since each plane of a complex sample has to be illuminated only once in order to get 3D information. Therefore, this method is most attractive in order to maintain viability in repetitive experiments of 3D cell biology or developmental biology. Focusing on laser light is also essential in Total Internal Reflection Fluorescence Microscopy (TIRFM) [20] of cell surfaces, in particular, if the angle of light incidence and therefore, the penetration depth of the evanescent electromagnetic field has to be varied (VA-TIRFM).

Lasers are often used for diagnostic purposes, e.g., OCT, fluorescence or Raman imaging. In this case, interactions with cells or tissue are supposed to be “reversible”, i.e., phototoxicity or any kind of damage is not expected to occur. This also holds to some degree for certain experiments of laser micro-manipulation using e.g., optical tweezers [21]. With increasing laser power further interactions occur, e.g., bio-stimulation, photochemical interactions (used e.g., in photodynamic therapy, PDT), as well as photo-thermal or optomechanical interactions (ablations). Here, not only the continuous wave (cw) laser power but also the duration of individual laser pulses play a predominant role.

The present paper focuses on various topics of laser-assisted techniques including 3D imaging, super-resolution imaging, spectral and fluorescence lifetime imaging as well as laser micromanipulation. Furthermore, some applications, e.g., fluorescence diagnosis, PDT (on a cellular level) and laser-assisted optoporation are emphasized. The manuscript describes these topics in a narrative way rather than giving a systematic and complete overview.

## 2. Laser-Assisted Methods

### 2.1. 3D Imaging

**Figure 1:**
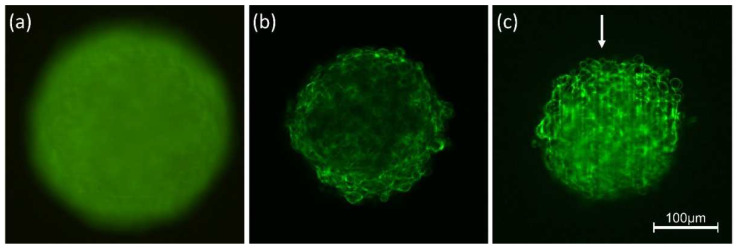
Spheroids of CHO-pAcGFP1-Mem cells recorded by conventional fluorescence microscopy (**a**), CLSM (**b**) and LSFM (**c**). Single planes are selected in (**b**,**c**) at a depth of 60 µm from the top of the spheroid; the arrow indicates the direction of light incidence in LSFM (excitation wavelength: 488 nm; fluorescence detected at λ ≥ 505 nm). Light intensity is reduced in the central part of the spheroid (**b**), and scattering increases along the light path (**c**). Reproduced from [30] with modifications.

Imaging of two-dimensional specimens, e.g., cell monolayers or biopsies of less than about 10 µm diameter, does not necessarily require a laser. However, cells are often cultivated in a three-dimensional environment, which in view of cell metabolism and physiology is closer to natural conditions. Conventional microscopy of 3D specimens often gives rather poor images with little information, as shown in Figure 1a for a Chinese Hamster Ovary (CHO) transfected with a membrane-associated Green Fluorescent Protein (GFP). Here, an image from the focal plane is superposed by out-of-focus fluorescence, and the total fluorescence is almost evenly distributed with very little structural information. Lasers can help to select images from individual cell layers, which are further combined in a well-resolved 3D image. One possibility is *Confocal Laser Scanning Microscopy (CLSM)* [22,23], where a laser beam is focused onto a diffraction limited spot within the sample, which is further imaged into a pinhole in front of the light (fluorescence) detector. Scanning of the laser beam over the sample gives a 2D image of a well-defined plane, and by moving the specimen in small steps in a vertical direction, numerous cell layers can be recorded, whose information is combined in a 3D image by appropriate software. Various CLSM methods with different recording velocities have been described, so far, including point scan and line scan microscopy as well as simultaneous illumination of various parts of a sample by Spinning-Disk Confocal Microscopy [24]. Lateral resolution is given in principle by the radius r = 0.61 λ/A_N_ (≥200 nm) of the diffraction limited spot (Airy disk) with λ corresponding to the laser wavelength and A_N_ to the numerical aperture of the microscope objective lens. If the pinhole, however, selects only part of the Airy disk (Airy scan [25] image scan [26], or re-scan microscopy [27]), this resolution can be improved by up to a factor of two. Laser Scanning Microscopy also includes Multiphoton Microscopy when ultra-short laser pulses are focused onto a small spot in the sample, thus exciting fluorescence by two or more photons and creating high contrast images without the need for any pinhole [28,29].

*Light Sheet Fluorescence Microscopy (LSFM)* [18,19] is another method of 3D microscopy, which uses wide-field techniques, but requires a laser beam, which is focused either into a light sheet (using a cylindrical lens) or into a diffraction limited spot, which is scanned along a line. In this case, optical excitation of the samples is perpendicular to the detection path, and special sample holders, e.g., micro-capillaries filled with liquid cultivation media or gels [31], are required. For 3D imaging, the light sheet and the microscope objective lens used for detection can be shifted simultaneously in an axial direction, so that the illuminated part of the sample is always in the focus of the objective lens. Both shifts may be different due to the refractive index of the immersion fluid, but this can be corrected either mechanically [32] or by software. Alternatively, the sample can be moved in an axial direction through a static light sheet. Similar to CLSM, z-stacks can be recorded with low fluorescence background and high contrast. Often the light sheet is generated by a low aperture lens (A_N_ ≈ 0.1), so that a large depth of focus L = nλ/A_N_^2^ ≤ 100 µm and a beam waist d = λ/A_N_ ≈ 5 µm are attained. This beam waist is sufficient for imaging single-cell layers of a multi-cellular specimen, while the detection lens provides the same lateral resolution as in conventional wide-field microscopy. Figure 1b,c shows fluorescence images of a single cell layer in a multi-cellular spheroid at a depth of 60 µm from its top recorded by CLSM or LSFM. While the lateral resolution is similar, the LSFM image shows strong attenuation in the central part of the sample due to light absorption and scattering. This attenuation is weaker for LSFM, but upon (mainly forward) scattering much contrast is lost, and some stripes are generated in the direction of light propagation. The main advantage of Light Sheet Microscopy (LSFM) over Confocal Microscopy is that only those planes are illuminated, which are recorded simultaneously so that light exposure is considerably lower than for those methods, where the recording of each image requires illumination of the whole specimen. Commercial light-sheet microscopes (e.g., Carl Zeiss, Olympus, Nikon), as well as open-source solutions or add-ons for existing microscopes, are presently available [32,33].

**Figure 2:**
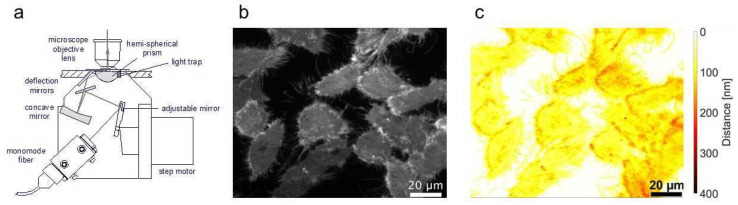
Microscope condenser for prism-based VA-TIRFM (schematic, (**a**)), TIRFM image of CHO-pAcGFP1-Mem cells incubated for 2 h with doxorubicin (2 µM) recorded at Θ = 66° (**b**), and cell-substrate topology in the range of 0–400 nm calculated from VA-TIRFM experiments (**c**); excitation wavelength: 476 nm, detection range: λ ≥ 490 nm.

In contrast to CLSM and LSFM, *Total Internal Reflection Microscopy (TIRFM)* [20] represents a method that is designed for studies of cell surfaces, in particular their plasma membranes. The method is based on the total internal reflection of a laser beam on a cell-substrate surface, thus generating an evanescent electromagnetic field that penetrates about 100 nm into the sample and permits selective excitation of membrane-proximal fluorophores. Two illumination concepts for TIRFM are reported in the literature: prism-type TIRFM, where light is incident on a cell layer via a glass prism, as shown in Figure 2a, and objective-type TIRFM, where laser light is focused close to the edge of the aperture of a high-aperture microscope objective lens. In both cases, the condition for the angle of light incidence Θ ≥ arcsin (n_2_/n_1_) has to be fulfilled with n_1_ corresponding to the refractive index of the glass substrate and n_2_ to that of the cell, namely the cytoplasm. As previously shown [34,35], variation of the angle of incidence (VA-TIRFM) permits the calculation of 3D cell-substrate topology with nanometer precision, e.g., upon application of cytotoxic or phototoxic agents. Furthermore, tumor cells and less malignant cells could be distinguished on the basis of prism-based VA-TIRFM [35]. Figure 2 shows a TIRFM image of Chinese Hamster Ovary (CHO) cells transfected with membrane-associated Green Fluorescent protein (GFP-Mem) incubated for 2 h with the cytostatic drug doxorubicin (2 µM, b) as well as cell-substrate topology calculated from a series of TIRFM images at 66° ≤ Θ ≤ 75° (c). More detailed experiments revealed that cell-substrate distances increased with the incubation time of doxorubicin. This may possibly be regarded as an early response to the application of this cytotoxic drug.

### 2.2. Super-Resolution Imaging

Resolution in microscopy is generally given by the Abbe criterion Δx ≥ λ /2A_N_ (for coherent light) or by the Rayleigh criterion Δx = 0.61 λ/A_N_ (for incoherent light, e.g., fluorescence). In both cases, the lateral resolution is restricted to about 200 nm. Only in the last 30 years have methods been developed, which permit to overcome this restriction, and which are summarized under the term “Super-resolution microscopy”. This term includes *Single Molecule Localization Microscopy (SMLM)* within a thin illuminated layer of a sample [36,37,38,39]. If a single molecule is detected n times, its localization can be determined with a precision of Δx = Δx_0_/√n with Δx_0_ corresponding to the Rayleigh criterion. Therefore, a precision of localization Δx = 20 nm results from n = 100 and Δx = 10 nm from n = 400. Illumination of thin layers most generally requires TIRFM or confocal techniques, but also light sheet illumination for tracking single molecules in living tissue has been reported [40]. Generally, SMLM techniques need an irradiance, which is about 100 times larger than the irradiance in conventional microscopy, as well as a prolonged exposure time of a few seconds up to minutes, so that the risk of phototoxic cell damage is very high.

Another super-resolution technique based on laser scanning microscopy is *Stimulated Emission Depletion (STED) Microscopy.* Here, the enhancement of resolution is due to suppression of fluorescence in the outer regions of a diffraction limited illumination spot by stimulated emission using a (second) donut-shaped laser beam. While a resolution of 30–70 nm can be achieved [41], the irradiance exceeds that of a conventional fluorescence microscope by a factor of 10^4^–10^5^ and may cause severe damage to living specimens. This problem was minimized with the introduction of MINFLUX nanoscopy, a technique based on the localization and tracking of single molecules in the intensity minimum of a donut-shaped laser beam. MINFLUX achieves nanometer resolution (isotropic: ≥2 nm) at moderate light exposure, which is comparable to a confocal laser scanning microscope [42].

A laser-based method with comparably low light exposure and an enhancement of resolution around a factor of two in comparison with the Abbe criterion is *Structured Illumination Microscopy (SIM)* [10,11]. Here, the sample is illuminated by two interfering laser beams creating a sinusoidal pattern that may be rotated to obtain isotropic resolution in a lateral plane. Images are recorded for at least three rotation angles and three phases (0, 2π/3, 4π/3) of the interference pattern, and a super-resolution image is calculated from a minimum of nine individual images. While two interfering laser beams are sufficient for enhanced resolution in the lateral dimension, three interfering beams are needed for increasing resolution in all three dimensions. It should be mentioned that resolution can be enhanced even by more than a factor of two, if the emission rate of the sample responds non-linearly to the illumination intensity, using e.g., saturation effects or photo-switching of fluorescent proteins [43].

In some cases, especially for larger specimens, *Lattice Light Sheet Microscopy,* a combination of Light Sheet Microscopy and SIM, is a useful tool for observing subcellular processes in three dimensions. This technique achieves a resolution around Δx = 150 nm and Δz = 280 nm at a high image acquisition speed, thus minimizing damage to cells due to phototoxicity [44].

**Figure 3:**
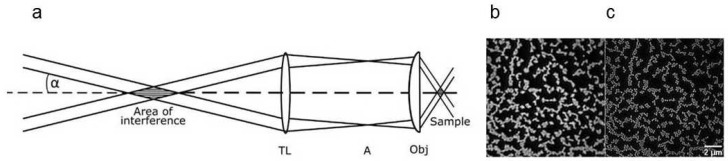
(**a**) Principle of Structured Illumination Microscopy (SIM) with interfering 1st diffraction orders of an optical grating or a spatial light modulator (SLM) in the focus of a collimating lens (for details of illumination see [45,46]). The interference pattern is imaged in the plane of the sample by the tube lens (TL) and the microscope objective lens (Obj) with an intermediate focus in the microscope aperture (A); Fluorescent polystyrene beads of 200 nm size recorded by wide-field microscopy (**b**) or SIM (**c**) with doubling of resolution. Reproduced from [46] with modifications.

The principle of SIM is depicted in Figure 3a; further details of a setup using a spatial light modulator (SLM) are described in [45,46]. Images of fluorescent polystyrene beads of 200 nm size recorded with wide-field microscopy and SIM are shown in Figure 3b,c. While the resolution of conventional wide-field microscopy is the same as the size of the beads, the SIM resolution is clearly better (about a factor of two) and allows individual beads to get well separated.

### 2.3. Spectral Imaging and Fluorescence Lifetime Imaging

**Figure 4:**
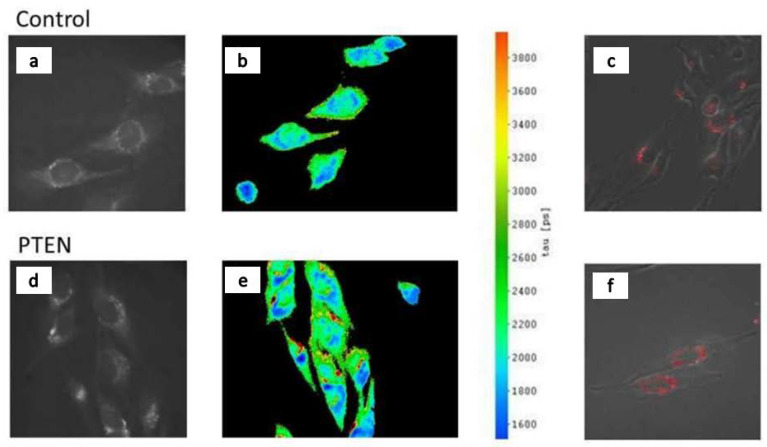
Autofluorescence images (**a**,**d**), fluorescence lifetime images including scale in picoseconds (**b**,**e**) and Raman (with superimposing phase contrast) images (**c**,**f**) of U251-MG glioblastoma cells (controls and after application of the tumor differentiating agent PTEN). Excitation wavelength: 375 nm (**a**,**b**,**d**,**e**), 514.5 nm (**c**,**f**); detection range: ≥420 nm (**a**,**b**,**d**,**e**), 535–550 nm (**c**,**f**). Excitation occurred by a quasi-continuous series of picosecond laser pulses (**a**,**b**,**d**,**e**) or by a continuous wave (cw) argon ion laser (**c**,**f**). Reproduced in parts from [51].

Besides spatial resolution, spectral or temporal data are often required in microscopy, e.g., to obtain information about the microenvironment or interactions of specific molecules inside a cell. Therefore, spectral imaging combines microscopy with an appropriate spectrometer or interferometer (for a review see [47,48]). Lasers are only required in special cases, e.g., Raman microscopy, when inelastic light scattering is measured at a wavelength λ, which is close to the excitation wavelength λ_0_ and permits the energy of a molecular vibration to be determined as ΔW = h c (1/λ_0_ − 1/λ), with h = 6.626 × 10^−34^ J s corresponding to Planck’s constant and c = 3.00 × 10^8^ m/s to the velocity of light. Since the intensity of inelastic light scattering is several orders of magnitude (typical factor: 10^6^–10^8^) lower than elastic light scattering, the latter is most commonly suppressed by a so-called Notch filter. Then, either spectral analysis or Raman imaging in a limited spectral range can be performed. In Figure 4c laser excitation occurs at λ_0_ = 514.5 nm, and after a Notch filter for this wavelength, Raman images are recorded at 535–550 nm.

Fluorescence Lifetime Imaging Microscopy (FLIM) [49,50] upon excitation with short laser pulses is another technique that provides additional information about molecular conformations or interactions with adjacent molecules. The lifetime τ of an excited molecular state, also termed “fluorescence lifetime”, corresponds to the reciprocal of its rates of deactivation k = k_F_ + k_NR_ + k_ET_, with k_F_ representing radiative (fluorescent) transitions, k_NR_ non-radiative transitions and k_ET_ energy transfer to adjacent molecules. Therefore, changes in the lifetime τ may reflect changes in molecular conformation or of the micro-environment of a molecule, singlet-triplet intersystem crossing (all via changes of k_NR_) or non-radiative energy transfer in the nanometer range. Figure 4 shows conventional autofluorescence images (a), fluorescence lifetime images (b) and Raman images (c) of U251-MG glioblastoma cells (controls and cells after application of the tumor differentiating agent PTEN, which is known to reduce the malignancy of the cells). Fluorescence images are dominated by the coenzyme nicotinamide adenine dinucleotide (NADH) [51] and are independent of the application of PTEN. In contrast, fluorescence lifetimes (again from NADH) appear slightly prolonged after the application of tumor differentiating agents, while Raman spectra are again quite similar. This suggests that fluorescence lifetimes (in addition to fluorescence spectra, not shown) may be an indicator of cell malignancy.

### 2.4. Laser Micromanipulation

**Figure 5:**
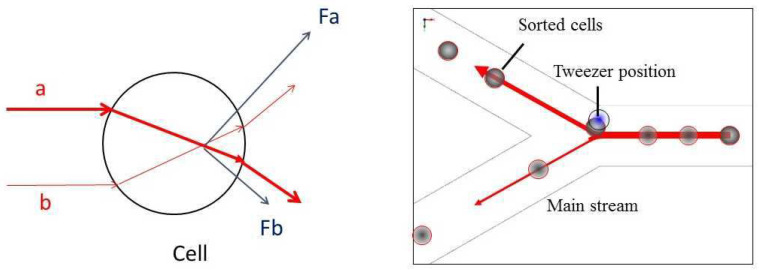
Principle of optical tweezers. Photons a (from the center of a laser beam) and photons b (from a peripheral part of the laser beam) are deflected by a transparent particle, e.g., a cell. The sum of repulsive forces F_a_ and F_b_ is directed towards the focus of the laser beam. Inset: Cell sorting experiment using an Nd:YAG-Laser at 1064 nm; cells are deflected from a main stream by optical tweezers.

So far, laser applications for microscopic imaging have been reported, where the wavelength λ of radiation or the photon energy W = h c/λ served as key parameters (with Planck’s constant h = 6.626 × 10^−34^ Js and the velocity of light c = 3.00 × 10^8^ m/s). However, photons also possess a momentum p = h/λ, which is rather small, but if a large number of photons is focused on a sub-micrometer laser spot, repulsive forces in the range of pico-Newtons to nano-Newtons are generated, which create a local pressure up to about 10^3^ N/m^2^ that is sufficient for moving particles such as cells or organelles. This is demonstrated in Figure 5 for the case that a large number of photons a (from the center of a laser beam) and a smaller number of photons b (from a peripheral part of the laser beam) are deflected by a transparent particle, e.g., a cell. This principle of optical trapping has been referred to as “optical tweezers” [21] with numerous applications dating back more than 30 years. Pilot applications include measurements of motility forces of cells [52], macromolecules [53] or organelles [21], micro-manipulation of cells or chromosomes [54], laser-assisted cell fusion [55], or sperm insertion into oocytes through a previously drilled hole [56]. Figure 5 shows an application of laser tweezers for cell sorting [57] using a specific channel structure on a glass chip. Cells are flowing in a main stream, but upon activation of an optical tweezer system by an appropriate trigger, they are deflected to a side channel, where they can be recovered and further analyzed. Cell survival in a laser trap—dependent on the laser wavelength and radiant exposure—is an important issue. Detailed studies are reported in the literature [58], but generally, for the far red or near-infrared spectral range, light exposures up to some hundred MJ/cm^2^ appear possible [59]. Since only very small areas of a cell are irradiated, these light doses are considerably higher than upon irradiation of whole cells.

## 3. Applications

### 3.1. Fluorescence Diagnosis/FRET

Fluorescence diagnosis is well established in experimental and clinical studies of cells, biopsies and living tissue. While intrinsic fluorescence has already found broad clinical application, fluorescence staining or transfection with fluorescent proteins is usually limited to studies of cell cultures with some diagnostic or pharmacological perspectives. Of particular interest is the method of Förster Resonance Energy Transfer (FRET) [15,16,17] between two molecules (intermolecular FRET) or between different chromophoric groups of a larger molecule, e.g., a protein (intramolecular FRET). This method is based on optical excitation of a so-called donor and interaction of optical transition dipoles with an acceptor, which is able to fluoresce, thus permitting to prove either molecular interactions or conformational changes of a molecule in the nanometer range. Due to the impact of chemical or pharmaceutical agents, the FRET technique is applied increasingly in biosensors and drug discovery systems [60,61,62,63]. FRET has been combined with TIRFM to detect selectively interactions within or close to the plasma membrane [60,64,65,66] as well as with light sheet microscopy (LSFM) to measure interactions within single layers of a 3D specimen [67,68].

**Figure 6:**
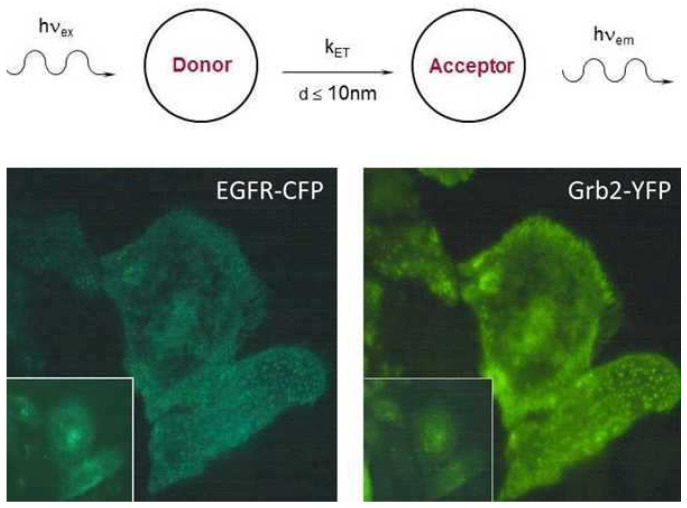
Principle of FRET from a donor to an acceptor molecule with the energy transfer rate k_ET_. TIRFM images of HeLa cells transfected with EGFR-CFP and Grb2-YFP encoding vectors in the emission ranges of the donor CFP (450–490 nm) and the acceptor YFP (λ ≥ 510 nm) upon FRET and possibly some additional direct excitation of the acceptor; excitation wavelength: 420–440 nm; image size: 60 µm × 60 µm. Insets: fluorescence images of the same object field after epi-illumination of whole cells (reproduced from [66] with modifications).

An example is given in Figure 6 showing the principle as well as an application of FRET from the Epidermal Growth Factor Receptor (EGFR) fused with Cyan Fluorescent Protein (CFP) to the growth factor receptor-bound protein 2 (Grb2) fused with Yellow Fluorescent Protein (YFP). If the fluorescent proteins are closer to each other than about 10 nm, non-radiative energy transfer occurs from the donor EGFR-CFP to the acceptor Grb2-YFP, and upon optical excitation of the donor, both donor and acceptor fluoresce. This is shown in Figure 6 for Total Internal Reflection Microscopy (TIRFM) and conventional wide-field microscopy (insets). Obviously, TIRFM enhances the signal/background ratio for this membrane-specific interaction. For quantitative evaluation, fluorescence spectra (ratio of acceptor/donor emission) as well as the fluorescence lifetime of the donor, which is shortened by FRET, can be used, as further described in [66]. FRET was probably stimulated in focal adhesions by the Epidermal Growth Factor (EGF) and possibly reduced by EGFR phosphorylation inhibitors. This opens the possibility to use this FRET sensor for a pharmacological test system, e.g., by replacing the fluorescence microscope with a multi-well reader system for simultaneous detection of a larger number of samples. In [66], a Total Internal Reflection (TIR) is described for a 96-well microtiter plate with a super-continuum picosecond fibre laser used for simultaneous excitation of all samples.

### 3.2. Photodynamic Therapy (PDT)

**Figure 7:**
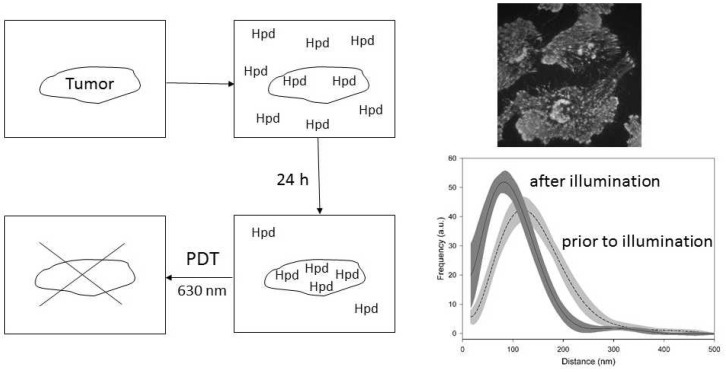
Photodynamic therapy (PDT): Principle of accumulation of a photosensitizer (here: Hpd) in a tumor and light-induced tumor destruction (left); membrane-associated 5-ALA-induced Protoporphyrin IX fluorescence in U373-MG glioblastoma cells assessed by TIRFM (upper right); cell-substrate topology prior and subsequent to illumination with red light (630 nm, 4 J/cm^2^), as evaluated from VA-TIRFM images at λ ≥ 590 nm. Median values ± median absolute deviations (MADs) were obtained from 10 independent experiments of cell-substrate topology each (lower right). Reproduced from [83] with modifications.

Owing to their tumor-localizing and photosensitizing properties, porphyrins such as hematoporphyrin and its derivatives (Hpd) as well as other chromophores (e.g., chlorines) have gained considerable interest in fluorescence detection and photodynamic therapy (PDT) of cancer [69]. While fluorescence results from a radiative transition from the excited singlet state S_1_ to the ground state S_0_ of photosensitizer molecules, PDT is due to intersystem crossing from S_1_ to the excited triplet state T_1_, from which the cytotoxic species singlet oxygen or superoxide radicals are generated. These species react with various biomolecules (e.g., proteins) and cause cell damage, preferentially after the application of red light. The principle of PDT is shown in Figure 7. After local or systemic application, a photosensitizer (e.g., Hpd) is first distributed all over the tissue but often accumulates within a tumor after a period of 6–24 h. This accumulation, which is a prerequisite for PDT, may be due to a tumor-specific micro-vasculature, pH effects or specific binding sites. Alternatively, 5-aminolevulinic acid (5-ALA), an intermediate in porphyrin biosynthesis, or related substances lead to an accumulation of protoporphyrin IX (PP IX) within tumor cells [70,71], possibly due to the reduced activity of the enzyme ferrochelatase, which converts PP IX to heme [72]. Meanwhile, PDT has been applied clinically in many disciplines including neurology [73], otolaryngology (ENT) [74,75], gastro-enterology [76,77], gynecology [78], urology [79] or dermatology [80], and tumors, as well as ocular or skin diseases (e.g., psoriasis [81]), were treated successfully using appropriate laser systems. In addition, laser irradiation proved to be efficient for fluorescence diagnosis or fluorescence-guided tumor resection, e.g., in neurosurgery [82].

Laser-assisted microscopy has been applied to cultivated cells in view of intracellular localization of photosensitizers or studies of their light-induced reactions such as photobleaching. Laser scanning microscopy is used for precision measurements, while time-resolved (sub-nano) detection methods in combination with picosecond laser excitation are used to distinguish different species, e.g., monomers, aggregates or ionic species, on the basis of their fluorescence lifetimes. Figure 7 proves the accumulation of 5-ALA-induced protoporphyrin IX by TIRFM in the plasma membrane of U373-MG glioblastoma cells, where it was revealed to be very sensitive to PDT [84]. This Figure also shows histograms of cell-substrate distances of PP IX in U373 MG glioblastoma cells prior to and subsequent to laser illumination (633 nm; 4 J/cm^2^), as evaluated from VA-TIRFM images at λ ≥ 590 nm [83]. A remarkable result is that upon application of this low, but for cellular PDT relevant light dose, cell-substrate distances decrease, indicating some increase in cellular adhesion and possibly some reduction of the metastatic potential, as further reported in [85].

### 3.3. Laser-Assisted Optoporation of Living Cells

**Figure 8:**
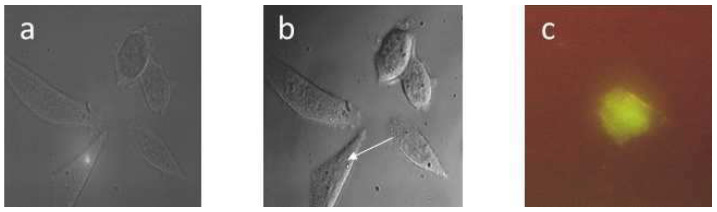
Chinese Hamster Ovary (CHO) cells (**a**) during laser irradiation, (**b**) immediately after irradiation (488 nm; 1 MW/cm^2^; 2.5 s) with the irradiated spot marked by an arrow, (**c**) fluorescence 24 after transfection with a GFP encoding plasmid. Image size: 100 µm × 100 µm (**a**,**b**), 60 µm × 80 µm (**c**). Reproduced from [91] with modifications.

In addition to laser trapping, e.g., in an optical tweezers system, laser microbeam techniques [86,87] can be used for microdissection, hole drilling or optoporation in order to make cell membranes penetrable for the uptake of small molecules or particles, e.g., fluorescent dyes or DNA plasmids used for cell transfection. Possible mechanisms—including photochemical, photothermal and optomechanical interactions (ablations)—are induced by continuous wave (cw) or pulsed lasers of different wavelengths, power and mode of operation. Photochemical reactions, used e.g., for PDT, generally need the lowest light doses, but only in individual cases can they be used for optoporation without risk of lethal damage [88,89], e.g., in the case of gene transfection or gene therapy. Often laser-assisted optoporation is associated with photothermal interactions, which is a certain range of light dosage proven to be reversible. This is demonstrated in Figure 8, where small spots of 1.0 µm diameter were irradiated by a cw argon ion laser at 488 nm and a light dose of 2.5 MJ/cm^2^ (applied during 2.5 s). After irradiation, tiny black spots—often surrounded by interference rings—could be seen, however, these disappeared within about five minutes [90,91]. At higher light exposure (≥5 MJ/cm^2^), permanent changes in morphology were observed, and this was concomitant with lethal damages, as evidenced by a colony formation assay [91]. The efficiency of laser-assisted transfection with a GFP encoding plasmid is demonstrated by the fluorescence image in Figure 8c. Transient changes, as observed in Figure 8 were related to an increase in temperature by a few degrees with a concomitant phase transition of membrane lipids from a rather rigid gel phase to a more fluid liquid crystalline phase [91], which may favor the uptake of certain molecules, e.g., fluorescence markers or DNA plasmids. Thus, the transfection rate of Chinese Hamster Ovary (CHO) cells was increased from about 5% to 15–30% due to laser-assisted optoporation using 40 µM phenol red (in cultivation medium) to enhance light absorption and rise of temperature. In further investigations, composite nanoshells [92] or magnetic carbon nanoparticles [93] were used as absorbers to create an appropriate heat profile for optoporation.

While cw lasers induced mainly thermal interactions, short-pulse (picosecond or femtosecond) lasers induced local ablation and transient opening of cell membranes so that exogenous material and even macromolecules could be introduced into living cells without photo-destructive effects. High repetition pulses from a mode-locked laser [94] as well as single near-infrared laser pulses were applied for this purpose [95]. An important step towards automation was the introduction of a continuous flow system, offering the prospect of high-throughput optoporation [96]. The use of femtosecond (instead of nano- or picosecond) lasers increased the optoporation rate due to multiphoton effects [97], and combination with plasmonic gold nanoparticles further enhanced the efficiency of optoporation due to an amplified localized electromagnetic field. Thus, a very high perforation rate of 70%, a transfection efficiency three times higher than for conventional lipofection and very low toxicity (<1%) were obtained [98].

The first step towards clinical application is represented by the delivery of impermeable substances into retinal explants after ultrafast laser microbeam-assisted injection [99]. In vivo optoporation of retinal ganglion cells (RGCs) targeted with functionalized gold nanoparticles was used to label these cells specifically with fluorescent conjugates. This provides a novel approach to selectively targeting retinal cells in diseased regions while sparing neighboring healthy areas [100]. Furthermore, local ablation and injury to individual cells by a laser microbeam were used to study the calcium metabolism around epithelial wounds [101].

It should be mentioned that laser-assisted optoporation has often been used in combination with a laser tweezer system, where cells or particles can be trapped and moved into the focus of a (second) laser beam for optoporation or further microbeam applications [102,103]. A laser microdissection and pressure catapulting technique (LMPC) has been developed for the characterization of single cells and their diverse biomolecules [104]. With LMPC, the force of focused laser light is utilized to excise selected cells or tissue areas from object slides, and after defocusing of the laser beam the sample is directly catapulted into an appropriate recipient vial. LMPC has been successfully applied to isolate and catapult cells from histological tissue sections, forensic material, as well as plant matter.

## 4. Conclusions

The unique properties of lasers—coherence, diffraction limited focusing, well-defined spectral properties and the possibility to create short light pulses—make them a valuable tool for imaging microscopy as well as for micro-manipulation. This improves the possibilities of 3D imaging, super-resolution imaging, spectral or fluorescence lifetime imaging and laser microbeam applications. While many relevant techniques are summarized, the applications described in this paper may be regarded as examples to demonstrate the potential of lasers in cell biology and—more generally—in several fields of biomedicine, e.g., ophthalmology, neurology and dermatology. Present and future development regarding, e.g., femtosecond lasers, super-continuum lasers or miniaturized laser diodes will further increase this potential.

## Figures and Tables

**Table 1:** Most convenient cw and pulsed lasers used for biomedical optics with data on wavelength, typical average power, pulse energy and pulse duration. Emission of laser pulses is either inherent or occurs after electro-optical (“Q-switch”) or acousto-optical modulation (“mode locking”). Femtosecond lasers (with pulse compression) are omitted.

Mode	Type	λ (nm)	P (W)	W_Imp_ (J)	t_Imp_ (s)
Cw laser (gas)	Ar^+^, Kr^+^	351–676	≤20		
	HeNe	543–633	≤0.1		
	CO_2_	10.6 µm	≤10^5^		
Cw laser (solid state)	Nd:YAG	532, 1064	≤300		
	Diode	≥370	≤1		
Pulse laser	Excimer	157–308	≤100	≤0.5	10^−8^
	Solid state (Nd^+^, Er^+^, Ho^+^)	532–2940	≤100	≤1	10^−4^
	Super-continuum fiber laser	400–2000	≤5	≤10^−7^	≤10^−11^
Q-Switch laser	Solid state (Nd:YAG)	256–1064	≤100	≤1	10^−8^
Modelocked laser	Ar^+^, dye, Nd:YAG	400–1000	≤10	≤10^−6^	10^−10^–10^−11^
	Ti: sapphire	325–1000	≤10	≤10^−6^	≤10^−11^

## Data Availability

Not applicable.
